# Comparative Study of Greater Palatine Nerve Block and Intravenous Pethidine for Postoperative Analgesia in Children Undergoing Palatoplasty

**Published:** 2009-12

**Authors:** Manjunath R Kamath, Sripada G Mehandale, Raveendra US

**Affiliations:** 1 Asst Prof, Department of Anaesthesiology and Critical Care, KS Hegde Medical Academy, Deralakatte, Mangalore, DK, 574018, Karnataka; 2 Assoc Prof, Department of Anaesthesiology and Critical Care, KS Hegde Medical Academy, Deralakatte, Mangalore, DK, 574018, Karnataka; 3 Prof & HOD, Department of Anaesthesiology and Critical Care, KS Hegde Medical Academy, Deralakatte, Mangalore, DK, 574018, Karnataka

**Keywords:** Greater palatine nerve block, Palatoplasty, Postoperative analgesia, Pethidine

## Abstract

**Summary:**

Greater palatine nerve block anaesthetizes posterior portions of the hard palate and its overlying soft tissues. This study compared the efficacy, safety, and ease of the nerve block for cleft palate surgeries in children with i.v. pethidine for postoperative pain management. A prospective, double blind, randomized trial, enrolled 50 children aged below 10 years scheduled for palatoplasty and were alternatively allocated to two groups. Group A received intravenous pethidine 1mg.kg^−1^, whereas Group B, bilateral greater palatine nerve block with bupivacaine 0.25%, 1ml on each side, before the surgical stimulation. Modified Aldrete Scoring System, Children's Hospital Eastern Ontario Pain Scale (CHEOPS) and Brussels Sedation Score were employed to assess recovery, quality of analgesia and sedation respectively, by the nursing staff. Whenever pain score was >8, 0.5mg.kg^−1^ of pethidine was given intravenously for rescue analgesia in both groups.

Recovery scores were better in Group B (p=0.007). In the immediate postoperative period, pain score was more in Group A (number of patients with pain score >8, 44% v/s 12%, p= .0117). Requirement for rescue analgesia was more in Group A (60 times v/s 7). The average sedation scores were similar. There was a higher incidence of agitation in Group A (66 vs. 30). The incidence of deep sedation was nearly half in Group B (34 Vs 63). Greater palatine nerve block was considered successful in 88% of cases. Greater palatine nerve block produces more effective, consistent and prolonged analgesia than pethidine.

## Introduction

Postoperative pain is expected but nonetheless undesirable ‘by-product’ of all surgical procedures. In adequate pain control may result in increased morbidity or mortality.^1^

Various studies world over have shown the tendency to under treat postoperative pain for the fear of resultant adverse effects like respiratory depression, nausea and vomiting, aspiration etc., due to overdose of analgesics. This is truer in case of young children undergoing surgeries. 2

As such, children with cleft palate tend to have a compromised airway due to associated congenital anomalies like Pierre Robin syndrome, Treacher Collin syndrome etc., After surgical correction of cleft palate (palatoplasty), they are more prone to develop postoperative respiratory difficulty due to narrowed airway, increased secretion, pain, and sedation caused by opioid analgesics.^3^,^4^ In recent times, undisputed supremacy of opioids in treating postoperative pain has been challenged by regional blocks mainly to overcome the side effects of narcotics. Several authors have attempted to validate effectiveness of regional blocks or peripheral nerve blocks to alleviate postoperative pain. Though a number of studies are available comparing systemic analgesics to regional blocks in treating postoperative pain due to herniotomies, circumcision, hydrocelectomy, hypospadias repair^5^, ophthalmic surgeries etc., surprisingly very few attempts have been made in employing nerve blocks for cleft surgeries.^6^,^7^

This study was done to compare quality of systemic analgesia achieved with intravenous pethidine and local analgesia with greater palatine nerve block for postoperative pain in children undergoing palatoplasty.

Greater palatine nerve supplies sensation to most of the hard palate. It exits through the greater palatine foramen. The nerve is blocked bilaterally as it exits through the foramen on palatal side opposite the anterior part of the 3^rd^ molar or posterior part of the 2^nd^ molar tooth. The foramen can be located by pressing a small cotton swab opposite first molar tooth and proceeding posteriorly till it ‘falls’ into a depression created by the foramen. A thin, short beveled needle is inserted from opposite side of the mouth at right angles into this depression and about 0.6 to 1ml of 0.25% bupivacaine is injected after negative aspiration for blood. 8

## Methods

After obtaining hospital ethics committee permission and informed written consent from the parent, 50 children scheduled for palatoplasty in Justice K.S.Hegde Charitable Hospital, Mangalore, between February 2003 and May 2004 were studied. Children belonging to American Society of Anesthesiologists (ASA) physical status Grade I and II and aged below 10 years were included in the study.

Children with co-morbid conditions like congenital heart disease, respiratory pathology, central nervous system disorders, associated other craniofacial anomalies like micrognathia, Pierre Robin Syndrome, Treacher Collins Syndrome were excluded from this study. Also children posted for combined procedures like palatoplasty with cheiloplasty or submucosal alveolar bone grafting (SABG) were not included. Known hypersensitivity to local anaesthetics or opioids was considered contraindication for inclusion in the study.

Preoperative visit was done to allay anxiety and to establish good rapport with the patients.

After clinical evaluation and routine investigations, all patients were premedicated with midazolam 0.5mg.kg^−1^ and atropine 1ml orally, one hour before surgery. After securing intravenous access and preoxygenation for 3 minutes, all children were induced with thiopentone 5mg.kg^−1^ and vecuronium bromide 0.1mg.kg^−1^ was used to facilitate intubation. Intubation was done with the appropriate sized endotracheal tube and ventilated with 33% oxygen in nitrous oxide and 0.4% halothane. Relaxation was maintained with vecuronium as and when required.

Patients were alternatively allocated to two groups, each consisting of 25 patients.

Group A: received intravenous pethidine 1mg.kg^−1^ and

**Fig 1:**
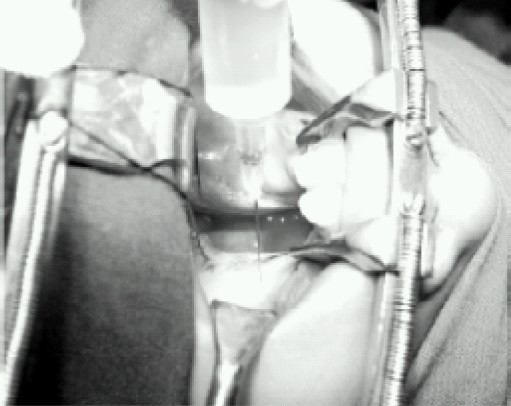
Local anaesthetic being into the greater palatine foramen

Group B: received bilateral greater palatine nerve block with bupivacaine 0.25%, usually 1ml on each side, before the surgical stimulation (Fig. 1). To minimize bleeding from the operative site, adrenaline diluted to 5mcg.ml^−1^ in normal saline was infiltrated. Recovery was assessed by employing Modified Aldrete Scoring System. (Table 1)^9^,^10^,^11^,^12^ In the postoperative ward, they received supplemental oxygen for one hour. Quality of analgesia was assessed by Children's Hospital Eastern Ontario Pain Scale (CHEOPS) (Table 2) as described by McGrath (1985).^13^Whenever the pain score is more than 8, rescue analgesia with 0.5mg.kg^−1^ of pethidine intravenously was given.

**Table 1:** Modified Aldrete Scoring System

Variable	Score	Interpretation
Activity	2	moves all extremities voluntarily/on command
	1	moves two extremities voluntarily/on command
	0	unable to move extremities voluntarily/on command
Respiration	2	able to breathe deeply and cough freely
	1	dyspneic, shallow breathing
	0	apneic
Circulation	2	able to breathe deeply and cough freely
	1	dyspneic, shallow breathing
	0	apneic
Consciousness	2	fully awake
	1	arousable on calling
	0	not responding
O_2_ Saturation	2	able to maintain O_2_ saturation more than 92% on room air
	1	supplemental O_2_ required to maintain SpO_2_>90%
	0	SpO_2_<90% with O_2_ supplementation

Maximum score= 10. Low score= equal or less than 6; High recovery score=7-10

**Table 2:** Children's Hospital Eastern Ontario Pain Scale(CHEOPS)

Item	Behavioral		Definition
Cry	No cry	1	Child is not crying
	Moaning	2	Child is moaning or quietly vocalizing silent cry
	Crying	2	Child is crying, but the cry is gentle or whimpering
	Scream	3	Child is in a full-lunged cry; sobbing; may be scored with complaint or without complaint
Facial	Composed	1	Neutral facial expression
	Grimace	2	Score only if definite negative facial expression
	Smiling	3	Score only if definite positive facial expression
Child Verbal	None	1	Child not talking.
	Other complaints	1	Child complains, but not about pain, e.g., I want to see mummy or I am thirsty
	Pain complaint	2	Child complaints about pain.
	Both complaints	2	Child complaints about pain and about other things, e.g., It hurts; I want my mummy
	Positive	0	Child makes any positive statement or talks about other things without complaint.0
Torso	Neutral	1	Body (not limbs) is at rest; torso is inactive.
	Shifting	2	Body is in motion in a shifting or serpentine fashion
	Tense	2	Body is arched or rigid.
	Shivering	2	Body is shuddering or shaking involuntarily
	Upright	2	Child is in a vertical or upright position
	Restrained	2	Body is restrained
Touch	Not touching	1	Child is not touching or grabbing at wound
	Reach	2	Child is reaching for but not touching wound.
	Touch	2	Child is gently touching wound or wound area
	Grab	2	Child is grabbing vigorously at wound.
	Restrained	2	Child's arms are restrained
Legs	Neutral	1	Legs may be e in any position but are relaxed; includes gently swimming or separatelike movements
	Squirm/ kicking	2	Definitive uneasy or restless movements in the legs and /or striking out with foot or feet.
	Drawn up/tensed	2	Legs tensed and /or pulled up tightly to body and kept there
	Standing	2	Standing, crouching or kneeling
	Restrained	2	Child's legs are being held down

Brussels Sedation Score^14^ (Table 3) was used to assess sedation. Along with these, heart rate, mean arterial blood pressure and oxygen saturation were assessed every 15 minutes for first 1 hour, then every 30 minutes for the next 2 hours, hourly thereafter up to 10 hours by blinded observer (staff nurse). Adverse effects of narcotics were looked for.

**Table 3:** Brussels Sedation Score

Score	Response
1	Unarousable
2	Responds to pain stimulation (trapezius muscle pinching but not to auditory stimulation)
3	Responds to auditory stimulation
4	Awake and calm
5	Agitated

Score: 1-2=oversedation,

3-4=correct sedation,

5=under sedation or agitation

### Statistical analysis:

The formula for calculating the sample size for qualitative study is

$$\begin{array}{c} n=\frac{4\mathrm{PQ}}{L^{2}} \end{array}$$

n=sample size, p=success rate of the block 95% (0.95)^8^

Q=1-P (1-0.95=0.05), L=allowable error, which we have taken as 10% using 95% confidence interval.

So n=(4×0.95×0.05)÷(0.1×0.1)=19

We included 25 children in each group to allow up to 30% dropout.

The results were analysed using chi-square test (χ^2^) and student's unpaired t test. A ‘p’ value of =0.05 was considered statistically significant, <0.01=highly significant (Hsig), <0.001=very highly significant (Vhsig) and >0.05=not significant (ns).

## Results

Both the groups were similar with respect to demographic characteristics and average duration of surgery. (Table 4)

**Table 4:** Demographic data

Criteria	Group A (n=25	Group B (n=25)	T
Age (years)	3.52±2.86	3.38±2.78	.1820
			p=0.857 ns
Weight (kgs)	11.66±4.87	11.68±5.10	1.400E-02
			p=0.989 ns
Sex (Male/Female)	11/14	14/11	c^2^=0.72
			P=0.396 ns
Duration of surgery (Min)	100.6±21.32	112.6±23.23	1.903
			p=.0631 ns
Average recovery Score	8.68±0.69	9.24±0.72	2.808
			p=.007 hsig

ns = not significant, hsg = highly significant (p value < 0.01)

### 1. Recovery score:

**Fig 2:**
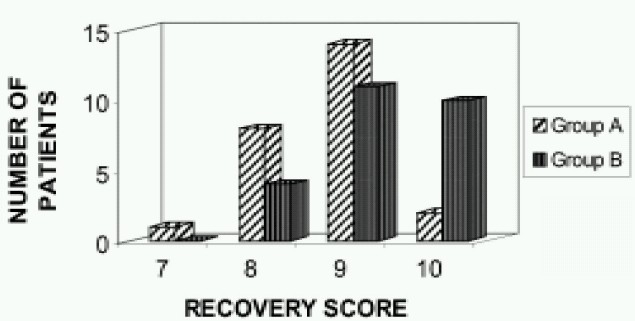
Recovery scores

Recovery was better in greater palatine nerve block group (p=0.007 as shown by the higher recovery scores in this Group B. i.e., 10 (40%) patients had maximum possible recovery score of 10 and none had recovery score of =7 (Fig. 2) . Whereas in Group A (pethedine), only 2(8%) patients had the maximum recovery score and one patient had a recovery score of 7.

### 2. Pain Scores:

**Fig 3:**
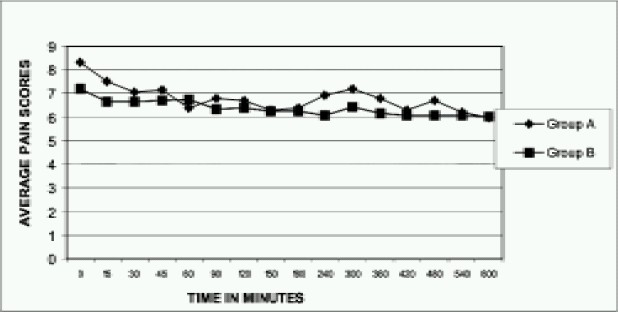
Average pain scores

**Fig 4:**
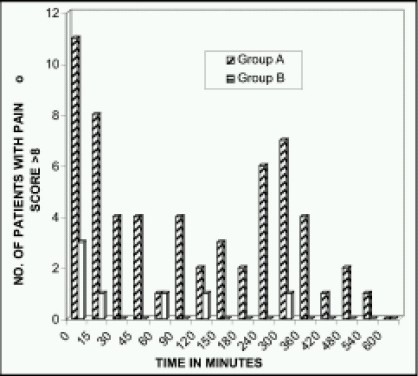
No. of patients having pain score more than 8

Average pain scores are almost similar and not statistically different between the two groups (Fig. 3). In the immediate postoperative period, 44% of Group A patients had pain score more than 8, whereas in Group B, it is only 12% which is statistically significant (p=0.0117) (Fig. 4). The total incidence of significant pain requiring rescue analgesia was 60 times in Group A, whereas it is only 7 in Group B i.e. almost 9 times higher. All the children in Group A required rescue analgesia at least once but 22 patients in Group B did not require any supplemental analgesia (Table 5). 80% of pethidine group patients needed rescue analgesia within the first hour of the postoperative period and among these, 32% needed rescue analgesia immediately in the post- operative ward. On an average, children in pethidine group (Group A) required rescue analgesia by 31.8 minutes. Considering the duration of intraoperative period, analgesia produced by pethidine in a dose of 1mg.kg^−1^ body weight did not last longer than 2-3hours. In general, every patient received about 2.4 injections of pethidine in the observation period.

**Table 5:** Rescue analgesia

Findings	Group A	Group B
Average doses of rescue analgesia per patient	2.4	0.28
Maximum number of dose per patient	5	3
Minimum number of dose per patient	1	0

In block group(Group B), only three patients required rescue analgesia throughout the study period. All these patients were given supplemental analgesia in the immediate postoperative period indicating inadequate or failed nerve block. The remaining 22(88%) patients did not require any additional analgesia for next 10 hours. This implies that the duration of analgesia produced by greater palatine nerve block with 0.25% bupivacaine is longer than ten hours.

### 3. Brussels Sedation Score

**Fig 5:**
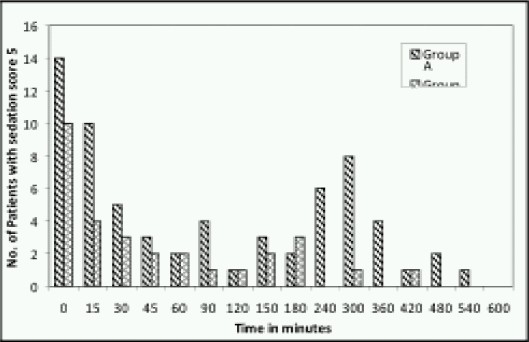
Number of patients with agitation

The average sedation scores are comparable. But there was higher incidence of agitation in Pethidine group (66 vs. 30) (Fig. 5). In Group A, all the patients had sedation score of 5 at least once in the observation period. In group B, numbers of patients with sedation score 5 were only 15 i.e., 60%. 40% of the Group B patients were comfortable throughout the observation period.

**Fig 6:**
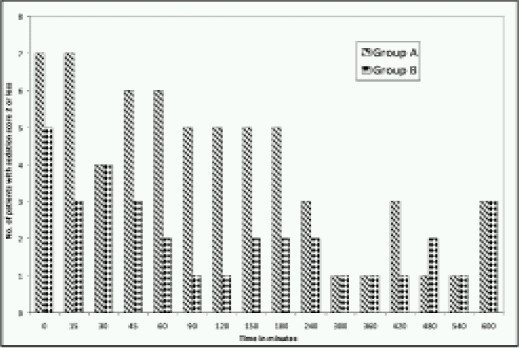
Number of patients with deep sedation

Incidence of oversedation (sedation score 2 or less) is more when pethidine was used (63 vs. 34) (Fig. 6). One patient was unarousable fifteen minutes after giving rescue analgesia. Fortunately he did not require any additional intervention to maintain adequate respiration. None of the patients with regional nerve block were unarousable.

### 4. Heart rate and Mean Arterial Blood Pressure

There is no significant difference in average heart rate and average mean arterial blood pressure among the groups. There were noticeable wide swings in the heart rate and mean arterial blood pressure among individual patients in pethidine group and in those who received rescue analgesia in block group. However, no such variation was observed among the patients in block group, who did not require any supplemental analgesia. Thus, the changes in heart rate and mean arterial blood pressure may be attributable to inadequate analgesia.

### 5. Hypoxia and other adverse events

Shivering was noticed in two patients belonging to block group which was managed conservatively.

There was an episode of airway obstruction in each of the groups, due to bleeding and secretions, which was relieved by suctioning and positioning the patient in left lateral. Bleeding in the postoperative ward was managed by packing and positioning. Other minor complications like anxiety, cough and secretion were self-limiting. However, Pethidine did not produce PONV and desaturation in children as frequently as reported. 15,16,17

## Discussion

Recently there is an increasing awareness regarding the need for complete well being of the child in the postoperative period and not just a pain free state. Sedation and other adverse events produced by opioids do not help in achieving such a goal. Local anaesthesia with nerve blocks appears to be the answer in such circumstances. Moreover, regional and general anaesthesia techniques are no longer considered as alternative but instead, as complementary. This is especially true in pediatrics where regional anaesthesia is essentially performed under general anaesthesia. The association of the two techniques has dramatically cut down the risks of both procedures.^15^,^16^ Hodges, Stephens and co-workers (2000)^7^ while reviewing anaesthesia for cleft surgeries opined that opioids are better avoided and intraoperative and postoperative analgesia can be achieved by local infiltration with local anaesthetics or by nerve block. But the authors have not mentioned about possibility of greater palatine nerve block for analgesia in this context.

Though majority of palatal mucosa is innervated by greater palatine nerve it also derives supply from lesser palatine and nasopalatine nerves, all are branches of maxillary nerve. During surgery, nasopalatine nerve is divided as it exits through the foramen. The lesser palatine nerve which supplies the soft palate lies in close proximity to greater palatine nerve in the canal before the latter comes out of the foramen.^8^ As we inject local anaesthetic into the foramen we might have blocked the maxillary nerve itself or its branches which may explain the total analgesia of the palate.

In our study, recovery from general anaesthesia is better when regional block is used for intra and postoperative analgesia. Our observation is in agreement with the findings of Bösenberg AT and Kimble FW(1995).^6^ They opined that postoperative awakening was rapid and smooth with no respiratory depression if regional analgesia is used instead of systemic opioids.

The total incidence of significant pain requiring rescue analgesia was 8.5 times lesser in the block group. This clearly demonstrates the opioid sparing effect of regional blocks as described by Schug and co-workers (1994)^17^. We used 0.25% concentration of the bupivacaine solution and the duration of analgesia outlasted observation period of 10 hours. 0.25% bupivacaine is known to produce prolonged peripheral nerve block. This is similar to the findings of Santhanam S and co-workers (2002)^18^ who reported that nearly half the children undergoing tympanomastoid surgery receiving greater auricular nerve block did not require additional analgesics in the first 24 hours. Heikey R and co-workers (1992)^19^ reported that the duration of analgesia ranged from 9.2 to 13 hours with the same concentration of bupivacaine for brachial plexus block. There is a need to extend the duration of period of observation to know the exact duration of analgesia produced by greater palatine nerve block with 0.25% bupivacaine. In our study, patients receiving pethidine were often deeply sedated or agitated at various points of time in the observation period whereas those who had greater palatine nerve block were mostly awake and calm, which is a desirable state. Our results support the views of Lewis N (1978).^20^ He commented that intraoperative nerve block greatly enhances postoperative pain relief. Current study demonstrates excellent postoperative analgesia achieved with greater palatine nerve block and there were no complications related to the technique. Success rate of block was 88% and it may be possible to improve this rate with experience. Alternatively, to minimize failures an open technique may be tried. That is, infiltrating the nerve under direct vision after the elevation of palatal flap, which is a part of surgery.

Drawback of this approach is there would be a lag period between the onset of surgical stimulation and nerve blockade. Further work is needed to evaluate suitability or supremacy of either of the techniques

Doyle E and Hudson I (1992)^3^ reported three cases of hypoxaemia among 143 cases of cleft palate repair. One case of respiratory depression was attributable to opioid analgesics and two cases had respiratory obstruction due to Pierre-Robin Syndrome. They also observed three cases of postoperative profound respiratory depression due to narcotics among children undergoing surgery for cleft lip. In contrast Bailey PL(1992)^21^observed that the postoperative hypoxaemia were more likely as a result of surgical technique than the use of opioids. Keith L 22 observed that palatoplasty by itself reduces the air passage and rarely, respiratory obstruction in the postoperative period sometimes requiring urgent airway interventions.

In the present study, despite the use of pethidine there was no incidence of desaturation in the postoperative period. Xue FS and co-workers (1998) 23 reported significant correlation between recovery scores and postoperative hypoxia. In their series, incidence of postoperative hypoxia was more than one-third of the children after palatoplasty when left on room air and 25% of the children had low recovery score (less than 7). Our data shows no incidence of hypoxia and all had recovery scores>7. They also came across high incidence of respiratory obstruction (upto 41%) in the postoperative period where as we had only 4%. This may be attributed to selection of patients without other congenital anomalies, shorter duration of surgery, difference in the surgical technique, better recovery from anaesthesia, routine supplemental oxygenation during the first hour of the postoperative period, adequate analgesia resulting in awake and calm child.

In our series, two children in block group had shivering, indicating antishivering property of pethidine. However, Pethidine did not produce PONV in children as frequently as reported. 24,25

Overall, there was definite advantage in the recovery and postoperative comfort in children received greater palatine nerve block.

**Conclusion:** Greater palatine nerve block is easy to learn and to perform with very high success rate. It is very effective in producing postoperative analgesia without any significant adverse events. Moreover it produces prolonged analgesia; duration of which has to be ascertained by further evaluation. By employing regional techniques we not only make the patient's stay in the postoperative period comfortable, but also avoid many serious adverse events associated with traditional analgesic regimens.
